# Waist circumference and insulin resistance: a community based cross sectional study on reproductive aged Iranian women

**DOI:** 10.1186/1758-5996-3-18

**Published:** 2011-08-10

**Authors:** Azita Zadeh-Vakili, Fahimeh R Tehrani, Farhad Hosseinpanah

**Affiliations:** 1 Obesity Research Center, Shahid Beheshti University of Medical Sciences, Tehran, Iran; 2 Reproductive Research Center, Research Institute for Endocrine Sciences, Shahid Beheshti University of Medical Sciences, Tehran, Iran

**Keywords:** Insulin resistance, Waist circumference, HOMA-IR, Cutoff, Iranian women

## Abstract

**Background:**

Although the positive relationship between insulin resistance (IR) and central obesity is well known, the direct relationship between waist circumference and IR is not clear yet and there is no consensus regarding the cut off value for waist circumference as a surrogate index for central obesity. The present study was aimed to determine the optimal cut-off value of waist circumference (WC) for predicting IR in reproductive aged Iranian women.

**Methods:**

Using the stratified, multistage probability cluster sampling method 1036 women were randomly selected from among reproductive aged women of different geographic regions of Iran. Following implementation of exclusion criteria, complete data for 907 women remained for analysis. Insulin resistance was evaluated by the homeostasis model assessment (HOMA-IR) and its cut off value was defined as the 95th percentile of HOMA-IR value for 129 subjects, without any metabolic abnormality. The optimal cut-off of WC in relation to HOMA-IR was calculated based on the receiver operating characteristics (ROC) curve analysis using the Youden index and the area under curve (AUC).

**Results:**

The mean age of the total sample of 907 subjects was 34.4 ± 7.6 years (range, 18 - 45 years). After adjustment for age the odds ratios (OR) of elevated HOMA-IR were progressively higher with increasing levels of waist circumference; the age adjusted OR of IR for women with WC > 95 cm in comparison to those subjects with WC < 80 cm, was 9.5 (95% CI 5.6-16.1). The optimal cutoff value for WC predicting IR was 88.5 cm; with a sensitivity and specificity of 71% and 64%, respectively.

**Conclusions:**

Waist circumference is directly related to insulin resistance and the optimal cut-off value for waist circumference reflecting insulin resistance is considered to be 88.5 cm for reproductive aged Iranian women.

## Background

Insulin resistance (IR) is the main pathophysiological feature of the metabolic syndrome (MetS), which in turn leads to increased risk of cardiovascular disease [1,2]. Central obesity, main diagnostic criteria for the MetS, is considered to predispose individuals for insulin resistance [3-5]. Waist circumference (WC); the best anthropometric indicator of central obesity [6,7], is closely associated with IR and provides a rapid, inexpensive and non-invasive way of identifying the presence of IR [8-11].

The International Diabetes Federation (IDF) has declared that waist is a gender and ethnic-group specific indicator and has adopted different cut-offs for waist circumference in different ethnicities [12,13]. The cut-off points for Euripides are 94 cm in men and 80 cm in women, while those for Chinese and South Asians are 90 cm in men and 80 cm in women [12]. For Iranians, based on both cross-sectional and longitudinal outcome based studies, the cut-off point of 95 cm for WC to diagnose MetS was identical in men and women [14,15]. However, these studies were primarily based on the relationship between waist circumference and risk factors for cardiovascular disease or multiple components of the metabolic syndrome other than insulin resistance [15-17] and there are a limited population-based studies for defining cut-off values of WC for diagnosis of IR [18-20]. Considering the lack of population based and sex specific data regarding optimal WC cut-off point for predicting IR in Iranians, we aimed to clarify the optimal cut-off point for diagnosis of insulin resistance, determined by homeostasis model assessment of insulin resistance (HOMA-IR), in a community based sample of healthy reproductive aged Iranian women.

## Methods

### Subjects

This cross sectional study was conducted in four randomly selected provinces of different geographic regions of Iran, i.e. Ghazvin (Central), Kermanshah (East), Golestan (North) and Hormozgan (South). A total of 1036 women, aged 18-45 years were selected using a stratified, multistage probability cluster sampling method. The frame for the selection of the sampling units was based on the Iranian household lists available in the Health Department. Menopausal women, those who had undergone hysterectomy or bilateral oophorectomy and pregnant women were excluded. A checklist questionnaire was completed at subjects' homes and eligible women were invited to a referral clinic in each province for a comprehensive interview and physical exam. Ultimately, data for 907 subjects remained in the final analysis that had completed questionnaire, physical and clinical exams.

Weight (kg) was measured while the patient was dressed in light clothing and without shoes, using digital scales and was recorded to the nearest 0.5 kg. Height was measured in a standing position, without shoes, using a measuring tape, while the shoulders were in a normal position and was recorded to the nearest 0.5 cm. Blood pressure was measured by a standard mercury sphygmomanometer with an appropriate sized cuff for arm diameter after 5 minutes rest and checked twice at an interval of at least 5 min. The mean value of these two measurements was used for the analyses. Waist was measured midway between the lower rib margin and the iliac-crest at the end of a gentle expiration. Body mass index was calculated as weight in kilograms divided by the height in meters squared (kg/m^2^).

An overnight fasting venous blood sample was obtained from each participant. Blood samples were collected in EDTA-treated test tubes. Plasma was separated in a refrigerated centrifuge at 3000 rpm for ten minutes. The sera were stored at -80°C until tested. Fasting serum glucose, triglycerides (TG), total cholesterol (TC) and high-density lipoprotein cholesterol (HDL-C) were measured using the enzymatic colorimetric method (Pars Azmon Inc., Tehran, Iran) by a Selectra 2 auto-analyzer (Vital Scientific, Spankeren, The Netherlands). The Friedewald equation was used to calculate low-density lipoprotein cholesterol (LDL- C); samples with TG greater than 400 mg/dl were assayed by a direct method. In all of these biochemical analyses, the inter- and intra-assay coefficients of variations were less than 2.5% and 3.2%, respectively. Insulin was assessed by the Immuno Enzyme Metric Assay (IEMA) (Mercodia, Uppsala, Sweden) and its intra- and inter-assay coefficients of variation were 2.4% and 5.8%, respectively. The ethical review board of the Research Institute for Endocrine Sciences approved the study proposal and informed consent was obtained from all subjects.

### Definitions

#### Insulin resistance was estimated by HOMA-IR according to the formula

HOMA-IR = [(Fasting serum insulin (μU⁄ L) × Fasting plasma glucose (mmol ⁄ L)] 22.5. Of the 907 subjects, insulin resistance cut-off value was 2.63 as determined using the 95th percentile of HOMA-IR of 129 study participants with BMI < 25 kg/m, non-diabetic (FBS < 126 mg/dl) and non-hypertensive (systolic blood pressure ≤ 130 mmHg, diastolic blood pressure ≤ 85 mmHg).

### Statistical analysis

Continuous variables were checked for normality using the one-sample Kolmogorov-Smirnoff test, and are expressed as mean ± standard deviation and/or median and interquartile ranges, as appropriate. The categorical variables are expressed as percentages. To assess the ability of WC to discriminate between women who were insulin resistant and those were not, receiver operating characteristics curve (ROC) was constructed and the area under the curve (AUC) was calculated. Using coordinates for drawing the ROC curve, the cut-off point for WC that had optimal values for sensitivity and specificity was calculated. We identified the optimal values for sensitivity and specificity as the ones that keep (1 - sensitivity)^2 ^+ (1 - specificity)^2 ^at minimum [21]. Data were analyzed using SPSS 15 statistical software (SPSS Inc., Chicago, IL).

## Results

**Figure 1:**
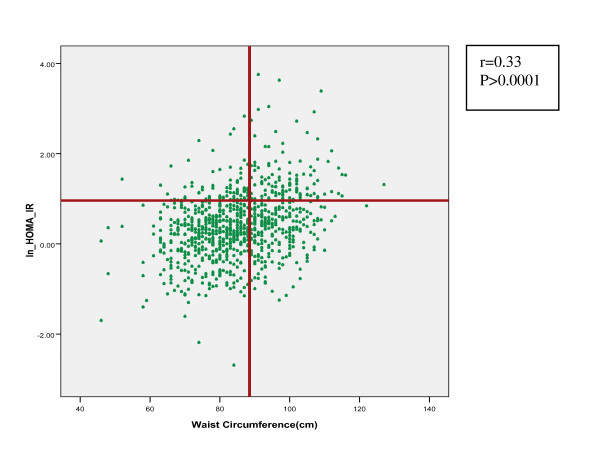
**Title Scatter plot of the relation between WC and HOMA-IR index in women**. The vertical line indicates the optimal WC cut-off point, derived from the ROC curve, for predicting IR (88.5 cm). The horizontal line delineates cases with IR (HOMA-IR ≥ 2.63).

Table 1 describes the characteristics of the study subjects. Age ranged between18-45 years, with a mean of 34.2 and body mass index (BMI) ranged between12.5-53.5 kg/m^2 ^with a mean of 26.9. Using the cut-off value 2.63 for identifying women with insulin resistant, there was 192 (21.2%) subjects categorized as the IR group. The characteristics and cardiovascular risks of women, with and without IR are demonstrated in Table 2. All of these risk factors except diastolic blood pressure were significantly higher among women with insulin resistance. The relationship between WC and HOMA-IR index is shown in Figure 1. There was a significant positive correlation between WC and HOMA-IR index (r = 0.32, p < 0.001).

**Table 1:** Characteristics of the study subjects (n = 907)

Variable	Value
	(means ± SD)
Age (Year)	34.4 ± 7.6
Systolic blood pressure (mmHg)	109 ± 14
Diastolic blood pressure (mmHg)	69.2 ± 11
Total cholesterol (mg/dl)	185 ± 42
LDL cholesterol (mg/dl)	111 ± 35
HDL cholesterol (mg/dl)	45 ± 13
Triglycerides (mg/dl)	142 ± 98
Weight (kg)	67.5 ± 12.8
Height (m)	159 ± 6
BMI (kg/m^2^)	26.9 ± 5.1
Waist (cm)	85.0 ± 12.2
Hip (cm)	105 ± 11
Fasting plasma glucose (mg/dl)	88.9 ± 26.1
Fasting plasma insulin (UI/ml)	9.0 ± 9.1
HOMA	2.1 ± 2.8
Insulin resistance(%)	21.2*

*Percent of women with insulin resistance.

**Table 2:** Characteristics of the study subjects according to the IR status (measured in terms of the HOMA value)

	With IR	Without IR	P-value
	(n = 192)	(n = 715)	
Age (Year)	34.9 ± 7.4	34.2 ± 7.7	0.256
Glucose (mg/dL)	106 ± 39	84.2 ± 18.2	< 0.001
Insulin (UI/mL)	19.1 ± 15.5	6.3 ± 2.7	< 0.001
HOMA	5.1 ± 5.1	1.29 ± 0.6	< 0.001
Total cholesterol (mg/dL)	200 ± 47	180 ± 39	< 0.001
LDL cholesterol (mg/dL)	120 ± 41	109 ± 33	< 0.001
HDL cholesterol (mg/dL)	39.5 ± 14.2	46.5 ± 12.2	< 0.001
Triglycerides (mg/dL)	201 ± 150	126 ± 70	< 0.001
Weight (kg)	74.4 ± 13.7	65.5 ± 11.8	< 0.001
Height (m)	158 ± 5	159 ± 6	0.799
BMI (kg/m2)	29.7 ± 5.6	26.1 ± 4.6	< 0.001
Waist (cm)	91.7 ± 12	83.1 ± 11.6	< 0.001
Hip (cm)	110 ± 12	103 ± 11	< 0.001
Systolic blood pressure (mmHg)	111 ± 14	108 ± 14	0.002
Diastolic blood pressure (mmHg)	70.4 ± 11.4	68.9 ± 10.9	0.087

Values are means ± SD.

The odds ratios (ORs) for having IR were increased according to WC categories. The age adjusted OR for having IR for women with WC > 95 cm in comparison to those subjects with WC < 80 cm, were 9.5 (95% CI 5.6-16.1) (Table 3).

**Table 3:** Age-adjusted odds ratios of elevated HOMA-IR according to categories of waist circumference

		Number of women (%)	Odds ratio (95% confidence interval)
		
Waist (cm)	Total	Elevated HOMA-IR*	Age-adjusted
< 80	344	31(9%)	1.0 (referent)
80-84	129	25(19.4%)	2.3 (1.3-3.9)
85-89	150	35(23.3%)	2.6 (1.6-4.2)
90-94	102	25(24.5%)	3.4 (2-5.8)
95 ≤	182	76(41.8%)	9.5 (5.6-16.1)

* The HOMA-IR ≥ 95% percentile (≥ 2.63)

**Figure 2:**
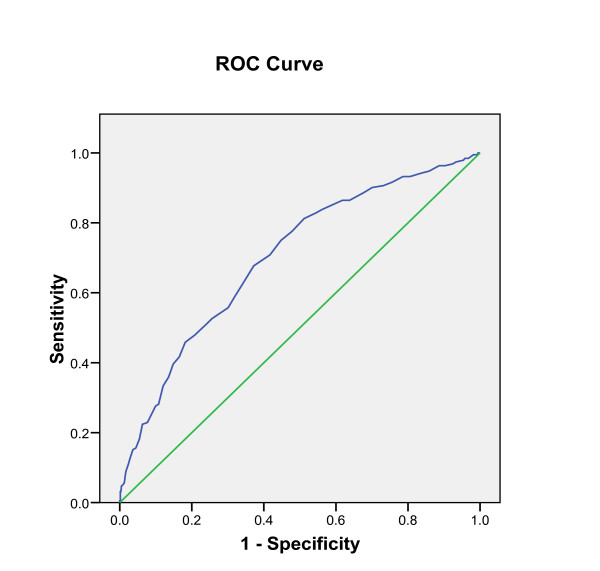
**Title: Receiver operating characteristics (ROC) curve for ability of the waist circumference to identify women with insulin resistance**. The area under curve (AUC) = 0.7 (0.63-0.78 CI 95%).

Figure 2 presents the ROC curves for the ability of the waist circumference to identify women with insulin resistance. Using the ROC curve analysis the optimal value for sensitivity and specificity that keep (1 - sensitivity) ^2 ^+ (1 - specificity) ^2 ^at minimum was 88.5 cm. Sensitivity and specificity were 71% and 64%, respectively.

## Discussion

In this population based cross sectional study we found that 88.5 cm is the optimal cut-off for predicting IR for reproductive aged Iranian women, our results indicating a significant, linear relationship between waist circumference and insulin resistance, measured by HOMA-IR. The odds ratio for the risk of insulin resistance using a cut-off < 80 cm for WC as a reference, increased progressively in proportion to the size of waist circumference.

Since insulin resistance is considered as an independent predictor for age related diseases, including cardiovascular disease, access to an accurate tool for measurement of the IR plays a vital role [22,23]. Although the euglycemic hyperinsulinemic clamp (Clamp-IR) is considered as the gold standard technique for estimation of insulin resistance, it is not applicable in epidemiologic studies. HOMA-IR, which is calculated from fasting plasma glucose (FPG) and insulin (FIRI), is highly correlated with the Clamp-IR; therefore it is a useful surrogate index of insulin resistance in both healthy and diabetic subjects [24-26]. Despite the wide use of HOMA-IR, no consensus has been reached regarding the HOMA-IR cut-off value for identifying subjects with IR. Lee et al. [27] and Radikova et al. [28] selected the 75^th ^percentile of non-diabetic population for cut-off point of IR which corresponded with HOMA-IR values of 3.04 and 2.29 respectively. However, Ascaso et al defined insulin resistance as a HOMA index > 3.8, corresponding to the 90th percentile of the distribution in a healthy adult Spanish population [29]. The threshold values of HOMA-IR to determine IR, in an Iranian population (aged 20-77 years), using the lower limit of the top quintile of HOMA-IR distribution values in normal subjects, was defined as 1.78 (1.69 for men and 1.81 for women); additionally, racial and ethnic variability in the HOMA-IR cut points to diagnose IR is probable [30,31]. Therefore, to implement the HOMA-IR method successfully, it is important to define specific cut-points for the race or age of the studied population. In the present study, a HOMA-IR value of 2.63, which corresponds to the 95th percentile of a population of healthy Iranian women, was chosen arbitrarily to define IR and to examine its relationship with WC. The prevalence of IR estimated in our study population was 21%, which is lower than that reported by Esteghamati (41.5%) [31], and can be explained by including younger and pre menopausal women in our study.

There is no consensus regarding the cut off value for waist circumference as a surrogate index for central obesity or as a component of metabolic syndrome, and this value is influenced by several factors including race, age, life style and reproductive status [13,32]. Several studies designed to determine the WC cut off values for diagnosing MetS in Iranian populations suggested a greater value in comparison to western populations [15-17], a higher value which may be explained by ethnic differences in body fat distribution, sedentary lifestyles and high carbohydrate diets and also the genetic tendency of Iranians to central obesity [33,34]. Nevertheless, there was no consensus regarding their suggested cut off value, which is influenced by the type of study or subject selection. The first national survey in an Iranian population of 3,024 adults revealed that the WC cut-offs for predicting at least two other components of the IDF-defined metabolic syndrome were 89 cm for men and 91 cm for women [15] while an outcome based cohort study suggested a WC cut-off of 94.5 [16]. Recently the Iranian National Committee of Obesity announced equal waist circumference cut-offs of ≥ 90 cm in both genders at risk for CVD risk factors, and that of ≥ 95 cm in both genders to be at high risk CVD events requiring immediate preventive measures [14].

The present study demonstrated that 88.5 cm is the optimal cut off for predicting IR for reproductive aged Iranian women, a value which was not changed considering the HOMA-IR values corresponding to the 90 and ≥ 97.5th percentile (2.31 and 3.65 respectively). Our proposed WC cutoff value is lower than those of the aforementioned studies for Iranian women. Considering the critical influence of aging on body fat distribution [35-38] this lower cut-off value may be explained by the fact that our study population is young, not yet influenced by menopausal status.

The main strength of the present study is its methodology, as it is a community based prevalence study carried out on an ethnically homogenous population, with an appropriate response rate of 90%. The amount of intra-assay variability in our data is also likely to be minimal because all the laboratory measurements were done at the same laboratory by the same person. The educational status and the prevalence of obesity in the present study was the same as that reported in a national study [39] and hence could justify and confirm our population as being representative of reproductive aged Iranian women. However as a main limitation we did not use gold standard technique for assessment of insulin resistance.

## Conclusions

In conclusion, our results revealed that 88.5 cm is the optimal cut off proposed for predicting IR for Iranian reproductive aged women. A comprehensive study for evaluation of the correlation between the values of HOMA-IR and Clamp-IR among reproductive aged women is suggested.

## Competing interests

The authors declare that they have no competing interests.

## Authors' contributions

AZ contributes in execution, analysis, manuscript drafting and critical discussion. FT contributes in study design, execution, analysis, manuscript drafting and critical discussion. FH contributes in analysis and manuscript drafting. All authors read and approved the final manuscript.

## References

[B1] Cornier MA Dabelea D Hernandez TL Lindstrom RC Steig AJ Stob NR Van Pelt RE Wang H Eckel RH The metabolic syndrome Endocr Rev 2008 29 777 822 10.1210/er.2008-0024 18971485 PMC5393149

[B2] Ferrannini E Haffner SM Mitchell BD Stern MP Hyperinsulinaemia: the key feature of a cardiovascular and metabolic syndrome Diabetologia 1991 34 416 422 10.1007/BF00403180 1884900

[B3] Saaristo TE Barengo NC Korpi-Hyovalti E Oksa H Puolijoki H Saltevo JT Vanhala M Sundvall J Saarikoski L Peltonen M Tuomilehto J High prevalence of obesity, central obesity and abnormal glucose tolerance in the middle-aged Finnish population BMC Public Health 2008 8 423 10.1186/1471-2458-8-423 19113993 PMC2628899

[B4] Lord J Thomas R Fox B Acharya U Wilkin T The central issue? Visceral fat mass is a good marker of insulin resistance and metabolic disturbance in women with polycystic ovary syndrome BJOG 2006 113 1203 9 10.1111/j.1471-0528.2006.00973.x 16753044

[B5] Peter G Kopelman IDC William H Dietz Clinical Obesity in Adults and Children 2009 3 Singapore: Wiley-Blackwell 512

[B6] Taylor RW Jones IE Williams SM Goulding A Evaluation of waist circumference, waist-to-hip ratio, and the conicity index as screening tools for high trunk fat mass, as measured by dual-energy X-ray absorptiometry, in children aged 3-19 y Am J Clin Nutr 2000 72 490 5 10919946 10.1093/ajcn/72.2.490

[B7] Rankinen T Kim SY Perusse L Despres JP Bouchard C The prediction of abdominal visceral fat level from body composition and anthropometry: ROC analysis Int J Obes Relat Metab Disord 1999 23 801 9 10.1038/sj.ijo.0800929 10490780

[B8] Lee JM Davis MM Woolford SJ Gurney JG Waist circumference percentile thresholds for identifying adolescents with insulin resistance in clinical practice Pediatr Diabetes 2009 10 336 42 10.1111/j.1399-5448.2008.00474.x 19175894

[B9] Rodriguez-Rodriguez E Palmeros-Exsome C Lopez-Sobaler AM Ortega RM Preliminary data on the association between waist circumference and insulin resistance in children without a previous diagnosis Eur J Pediatr 2011 170 35 43 10.1007/s00431-010-1260-1 20676898

[B10] Göran N Pär H Tommy J Ingemar L Åke T Rosanne F John Ö Waist circumference alone predicts insulin resistance as good as the metabolic syndrome in elderly women European journal of internal medicine 2008 19 520 526 10.1016/j.ejim.2008.01.018 19013381

[B11] Wahrenberg H Hertel K Leijonhufvud BM Persson LG Toft E Arner P Use of waist circumference to predict insulin resistance: retrospective study BMJ 2005 330 1363 4 10.1136/bmj.38429.473310.AE 15833749 PMC558285

[B12] Alberti K Zimmet P Shaw J The metabolic syndrome--a new worldwide definition Lancet 2005 366 1059 1062 10.1016/S0140-6736(05)67402-8 16182882

[B13] Alberti K Eckel R Grundy S Zimmet P Cleeman J Donato K Fruchart J James W Loria C Smith S Harmonizing the metabolic syndrome: a joint interim statement of the International Diabetes Federation Task Force on Epidemiology and Prevention; National Heart, Lung, and Blood Institute; American Heart Association; World Heart Federation; International Atherosclerosis Society; and International Association for the Study of Obesity Circulation 2009 120 1640 1645 10.1161/CIRCULATIONAHA.109.192644 19805654

[B14] Azizi F Hadaegh F Khalili D Esteghamati A Hosseinpanah F Delavari A Larijani B Mirmiran P Zabetian A Mehrabi Y Kelishadi R Aghajani H Appropriate definition of metabolic syndrome among Iranian adults: report of the Iranian National Committee of Obesity Arch Iran Med 2010 13 426 8 20804311

[B15] Delavari A Forouzanfar MH Alikhani S Sharifian A Kelishadi R First nationwide study of the prevalence of the metabolic syndrome and optimal cutoff points of waist circumference in the Middle East: the national survey of risk factors for noncommunicable diseases of Iran Diabetes Care 2009 32 1092 7 10.2337/dc08-1800 19279302 PMC2681035

[B16] Hadaegh F Zabetian A Sarbakhsh P Khalili D James WP Azizi F Appropriate cutoff values of anthropometric variables to predict cardiovascular outcomes: 7.6 years follow-up in an Iranian population Int J Obes (Lond) 2009 33 1437 45 10.1038/ijo.2009.180 19752876

[B17] Mirmiran P Esmaillzadeh A Azizi F Detection of cardiovascular risk factors by anthropometric measures in Tehranian adults: receiver operating characteristic (ROC) curve analysis Eur J Clin Nutr 2004 58 1110 8 10.1038/sj.ejcn.1601936 15280905

[B18] Sumner A Sen S Ricks M Frempong B Sebring N Kushner H Determining the waist circumference in african americans which best predicts insulin resistance Obesity (Silver Spring) 2008 16 841 846 10.1038/oby.2008.11 18292752

[B19] Tulloch-Reid M Ferguson T Younger N Van den Broeck J Boyne M Knight-Madden J Samms-Vaughan M Ashley D Wilks R Appropriate waist circumference cut points for identifying insulin resistance in black youth: a cross sectional analysis of the 1986 Jamaica birth cohort Diabetology & Metabolic Syndrome 2010 2 68 10.1186/1758-5996-2-68 21134291 PMC3017019

[B20] Tabata S Yoshimitsu S Hamachi T Abe H Ohnaka K Kono S Waist circumference and insulin resistance: a cross-sectional study of Japanese men BMC Endocr Disord 2009 9 1 10.1186/1472-6823-9-1 19138424 PMC2635363

[B21] Akobeng AK Understanding diagnostic tests 3: Receiver operating characteristic curves Acta Paediatr 2007 96 644 7 10.1111/j.1651-2227.2006.00178.x 17376185

[B22] Yip J Facchini FS Reaven GM Resistance to insulin-mediated glucose disposal as a predictor of cardiovascular disease J Clin Endocrinol Metab 1998 83 2773 6 10.1210/jc.83.8.2773 9709945

[B23] Facchini FS Hua N Abbasi F Reaven GM Insulin resistance as a predictor of age-related diseases J Clin Endocrinol Metab 2001 86 3574 8 10.1210/jc.86.8.3574 11502781

[B24] Ntyintyane L Panz V Raal F Gill G Comparison between surrogate indices of insulin sensitivity and resistance, and the hyperinsulinaemic euglycaemic glucose clamp in urban South African blacks with and without coronary artery disease Diab Vasc Dis Res 2010 7 151 7 10.1177/1479164109360271 20382779

[B25] Yokoyama H Emoto M Fujiwara S Motoyama K Morioka T Komatsu M Tahara H Koyama H Shoji T Inaba M Nishizawa Y Quantitative insulin sensitivity check index and the reciprocal index of homeostasis model assessment are useful indexes of insulin resistance in type 2 diabetic patients with wide range of fasting plasma glucose J Clin Endocrinol Metab 2004 89 1481 4 10.1210/jc.2003-031374 15001651

[B26] Chen CN Chuang LM Wu YT Clinical measures of physical fitness predict insulin resistance in people at risk for diabetes Phys Ther 2008 88 1355 64 10.2522/ptj.20080064 18801854

[B27] Lee S Choi S Kim HJ Chung YS Lee KW Lee HC Huh KB Kim DJ Cutoff values of surrogate measures of insulin resistance for metabolic syndrome in Korean non-diabetic adults J Korean Med Sci 2006 21 695 700 10.3346/jkms.2006.21.4.695 16891815 PMC2729893

[B28] Radikova Z Koska J Huckova M Ksinantova L Imrich R Vigas M Trnovec T Langer P Sebokova E Klimes I Insulin sensitivity indices: a proposal of cut-off points for simple identification of insulin-resistant subjects Exp Clin Endocrinol Diabetes 2006 114 249 56 10.1055/s-2006-924233 16804799

[B29] Ascaso JF Romero P Real JT Lorente RI Martinez-Valls J Carmena R Abdominal obesity, insulin resistance, and metabolic syndrome in a southern European population Eur J Intern Med 2003 14 101 6 10.1016/S0953-6205(03)00022-0 12719027

[B30] Nakai Y Nakaishi S Kishimoto H Seino Y Nagasaka S Sakai M Taniguchi A The threshold value for insulin resistance on homeostasis model assessment of insulin sensitivity Diabet Med 2002 19 346 7 10.1046/j.1464-5491.2002.00712_3.x 11943012

[B31] Esteghamati A Ashraf H Esteghamati AR Meysamie A Khalilzadeh O Nakhjavani M Abbasi M Optimal threshold of homeostasis model assessment for insulin resistance in an Iranian population: the implication of metabolic syndrome to detect insulin resistance Diabetes Res Clin Pract 2009 84 279 87 10.1016/j.diabres.2009.03.005 19359063

[B32] Azadbakht L Esmaillzadeh A Dietary and non-dietary determinants of central adiposity among Tehrani women Public Health Nutr 2008 11 528 34 17764604 10.1017/S1368980007000882

[B33] Kelishadi R Alikhani S Delavari A Alaedini F Safaie A Hojatzadeh E Obesity and associated lifestyle behaviours in Iran: findings from the First National Non-communicable Disease Risk Factor Surveillance Survey Public Health Nutr 2008 11 246 51 17625028 10.1017/S1368980007000262

[B34] Nabipour I Amiri M Imami SR Jahfari SM Nosrati A Iranpour D Soltanian AR Unhealthy lifestyles and ischaemic electrocardiographic abnormalities: the Persian Gulf Healthy Heart Study East Mediterr Health J 2008 14 858 68 19166169

[B35] Stevens J Katz EG Huxley RR Associations between gender, age and waist circumference Eur J Clin Nutr 2010 64 6 15 10.1038/ejcn.2009.101 19738633 PMC5909719

[B36] Ebrahimi-Mameghani M Scott JA Der G Lean ME Burns CM Changes in weight and waist circumference over 9 years in a Scottish population Eur J Clin Nutr 2008 62 1208 14 10.1038/sj.ejcn.1602839 17622259

[B37] Barbieri M Rosaria Rizzo M Manzella D Paolisso G Age-related insulin resistance: is it an obligatory finding? The lesson from healthy centenarians Diabetes/Metabolism Research and Reviews 2001 17 19 26 10.1002/dmrr.178 11241888

[B38] Lahti-Koski M Harald K Mannisto S Laatikainen T Jousilahti P Fifteen-year changes in body mass index and waist circumference in Finnish adults Eur J Cardiovasc Prev Rehabil 2007 14 398 404 10.1097/HJR.0b013e32800fef1f 17568239

[B39] Janghorbani M Amini M Willett WC Mehdi Gouya M Delavari A Alikhani S Mahdavi A First nationwide survey of prevalence of overweight, underweight, and abdominal obesity in Iranian adults Obesity (Silver Spring) 2007 15 2797 808 10.1038/oby.2007.332 18070771

